# Provider competence in hypertension management and challenges of the rural primary healthcare system in Sichuan province, China: a study based on standardized clinical vignettes

**DOI:** 10.1186/s12913-022-08179-9

**Published:** 2022-07-01

**Authors:** Yuju Wu, Ruixue Ye, Qingzhi Wang, Chang Sun, Sha Meng, Sean Sylvia, Huan Zhou, Dimitris Friesen, Scott Rozelle

**Affiliations:** 1 Department of Health Behavior and Social Science, West China School of Public Health and West China Fourth Hospital, Sichuan University, No. 16, Section 3, South Renmin Road, Chengdu, 610041 Sichuan People’s Republic of China; 2 Department of Operation Management, West China Hospital, Sichuan University, Chengdu, 610041 Sichuan China; 3 Department of Health Policy and Management, Gillings School of Global Public Health, University of North Carolina at Chapel Hill, Chapel Hill, North Carolina USA; 4 Freeman Spogli Institute for International Studies, Stanford University, California, Stanford USA

**Keywords:** Provider’s competence, Standardized clinical vignettes, Healthcare system, Hypertension, Rural China

## Abstract

**Background:**

Improving primary care providers’ competence is key to detecting and managing hypertension, but evidence to guide this work has been limited, particularly for rural areas. This study aimed to use standardized clinical vignettes to assess the competence of providers and the ability of the primary healthcare system to detect and manage hypertension in rural China.

**Methods:**

A multi-stage random sampling method was administered to select target health facilities, providers, and households. The clinical vignette script was developed to evaluate provider competence in managing first-visit patients with symptoms of hypertension. Logistic regression was used to explore the factors correlated with provider competence. Provider referral and management rates were combined with patients’ facility sorting behaviors to assess the ability of the rural healthcare system to manage hypertension in three policy scenarios.

**Results:**

A total of 306 providers and 153 facilities were enrolled in our study. In the 306 clinical vignette interactions, 25.9% of providers followed the national guidelines for hypertension consultation. The correct diagnosis was achieved by only 10.1% of providers, and 30.4% of providers were able to prescribe the correct treatment. Multi-variable regression results showed that younger providers (OR = 0.85, 95%CI: 0.73, 0.98) and those who work in township health centers (OR = 4.47, 95%: 1.07, 18.67) were more likely to provide a correct diagnosis. In a free-selection scenario, 29.8% of patients with hypertension were managed correctly throughout the rural system. When all patients first visit village clinics, system-level correct management is reduced to 20.5% but increases to 45.0% when all patients first visit township health centers.

**Conclusions:**

Rural primary care providers do not have enough competence to detect and treat hypertension cases in China to an acceptable degree. Policy constraints may limit the competence of the rural healthcare system. Research to improve detection and treatment competence in hypertension and optimize health policy is needed.

**Supplementary Information:**

The online version contains supplementary material available at 10.1186/s12913-022-08179-9.

## Introduction

Hypertension is the leading global risk factor for early death and disability and is the primary cause of death in China [1]. From 1990 to 2016, China had the highest number of deaths caused by high blood pressure of any country, ranging from 1.4 million to 2.3 million [2]. In 2016, nearly 50% of adults in China had hypertension, of which less than 10% of cases were well controlled [3]. The risk is especially severe in rural areas, which often have a higher hypertension prevalence and lower awareness, treatment, and control than do urban areas [3].

The healthcare system plays a foundational role in dealing with the increasingly high prevalence of noncommunicable diseases [4], including hypertension. During the nation’s health system reform, China has moved toward an “integrated care” model that promotes a system in which patients are encouraged to make their initial visit to primary care providers in local facilities [5]. China’s rural health system includes three tiers of providers: Village clinics (VCs), township health centers (THCs), and county hospitals (CHs). VCs and THCs represent the bottom two tiers in the healthcare system [6]. Under China’s guidelines for hypertension management, providers from VCs and THCs take primary responsibility in diagnosing patients during their first visit as well as managing diagnosed patients [7]. Although patients are free to choose among any of the three tiers for hypertension care without a referral, first-visit patients typically receive a referral from their primary care providers in VCs and THCs to visit VCs, then THCs, and, finally, CHs if their health issues have not been resolved during their previous visits.

One of the fundamental problems with making VCs and THCs the first point of contact for hypertension patients is that the primary care providers in these facilities often have limited knowledge of prevention, detection, diagnosis, and treatment of hypertension [8, 9]. To address this problem, China’s government issued policies and guidelines for hypertension management and control to guide the clinical practices of providers in a primary healthcare setting [10]. For example, in 2009, the Chinese central government launched the National Essential Public Health Services (NEPHS) to improve the accessibility for the Chinese population of essential public health services and strengthen the role and competence of primary health care providers in the prevention and management of chronic diseases including hypertension [11].

However, provider competence in detecting and managing hypertension in rural China has to date been underexplored in the academic literature. Most research on hypertension in rural China has focused on the prevalence of the disease and its risk factors, patient awareness of the disease, and associated complications [9, 12–14]. Research has evaluated the ability of VCs and THCs to detect and manage other diseases in rural China [15], however, few studies have examined how hypertension patients are detected, treated, and managed when visiting these tiers of primary care providers, despite their importance in managing hypertension under NEPHS. There is also little known about the differences in health system competence in relation to patient’ facility sorting behaviors (patients’ initial choice to visit VCs versus THCs). This is particularly relevant given the gaps in healthcare quality between different tiers of providers. Given the large emphasis on primary care providers in managing hypertension, understanding provider competence and how it may vary by patients’ visiting behaviors is vital to improving hypertension management in China.

There are also methodological limitations in previous studies of hypertension management. Most hypertension management studies in both the international literature and in China have used survey-based questionnaires to assess provider competence [16–20]. Although this approach can assess providers’ knowledge and adherence to national diagnosis or treatment criteria [19, 20], surveys can often fail to measure a provider’s competence to detect and manage hypertension in practice. Clinical vignettes using simulated medical conditions are a cost-effective and easily-administered approach to evaluate provider competence [21, 22]. Although clinical vignettes do not necessarily represent actual clinical practice, this approach has been validated for measuring the management of a wide variety of conditions and can reflect providers’ actual behavior [23] and can provide an opportunity to observe and measure provider competence in the detection and management of hypertension. Unfortunately, few previous studies have used clinical vignettes to evaluate competence in hypertension detection and management [24, 25], resulting in a gap in the literature.

To address these gaps in the literature, this study aims to use standardized clinical vignettes to assess the competence of rural providers and the ability of the rural healthcare system to detect and manage hypertension in rural China. To meet this overall goal, we have the following three objectives. First, we examine the competence of rural detection and management of patients symptomatic of hypertension at the provider level. Second, we explore the correlates of hypertension detection and management at facility and provider levels. Third, we combine patients’ facility sorting behaviors and provider-level competence in three policy scenarios to analyze the competence of the rural healthcare system in managing hypertension.

## Methods

### Setting and sampling

The facilities, providers and households in this study were sampled from rural areas in one prefecture of Sichuan Province in Western China. Nearly half (47.7%) of Sichuan’s population lives in rural areas. As of 2021, there are in total of 4317 THCs and 54,202 VCs in the province, and with an average of 26 providers in each THC, and 1.4 providers in each VC [26].

#### Facilities and providers

The facilities and providers in this study were sampled from rural areas in one prefecture of Sichuan Province in Western China using a four-stage random sampling protocol (Additional Fig. 1). First, five counties were randomly selected from the six counties in the prefecture. Second, ten townships were randomly selected in each sampled county, totaling 50 townships. All THCs in the sample townships were enrolled in the study. Third, two villages were randomly selected from each sample township, and all VCs in the sample villages were enrolled. Three sample villages had two VCs, while the remaining villages had only one. This led us to a final sample of 153 facilities, including 50 THCs and 103 VCs. All providers of general and internal medicine who were on duty on our survey day from the sample facilities were surveyed, totaling 306 providers. About 35.9% of providers were from VCs, which made up 67.3% of facilities.

#### Households

To measure patient facility sorting behaviors and the outcomes of the initial visit, we collected initial facility choices from patients with symptoms of hypertension at a household level. The facility sorting behavior of patients (i.e., choice of which facility a patient visits first) is important in evaluating the ability of the healthcare system to manage hypertension, as this determines how patients are treated, which subsequent facilities they visit, and the efficacy of the treatment process. Ten households were randomly selected from the resident roster of each sample village, and five households were randomly selected from the non-communicable disease (NCD) roster of each sample village. In total, 15 households were enrolled into our study from each sample village.

### Data collection

#### Facility and provider survey

Structured questionnaires were administrated to collect facility and provider information. Trained investigators interviewed providers to obtain their age, gender, medical education, qualifications, medical experience, income, and medical training. Investigators also assessed providers’ work motivation using the Motivation at Work Scale, as providers’ motivation to work has found to correlate to healthcare quality [27]. Facility information, including patient volume, the number of full-time staff, and the number of hypertension patients, was collected from the head staff member in charge of each facility.

#### Household survey

Patient-level demographics, including household socio-demographic characteristics, whether a member of the household had been diagnosed with hypertension, and facility sorting behaviors, were collected by trained investigators in a face-to-face interview survey. Facility sorting behaviors included which facility a household member chose to visit initially when they had symptoms related to hypertension, such as a headache or dizziness.

#### Standardized clinical vignettes

Standardized clinical vignettes were used to measure provider competence in the consultation process, which included the provider’s ability to correctly diagnose and manage a patient with typical symptoms of hypertension. A standardized case script was developed by our research team following several rounds of consultation with hypertension specialists. Local hypertension prevention and management authorities also were included in the consultation to adapt the script to a local context.

During the survey, two enumerators presented the vignette to providers. The detailed process of conducting the clinical vignettes can be found in Supplementary file 1. Based on the documentation of the clinical vignette interaction, provider competence was evaluated in three domains: 1) the consultation process, determined by the average percentage of recommended questions and examinations (ARQE); 2) correct diagnosis, determined by the accuracy and completeness of the diagnosis; and 3) correct treatment, determined by the overall accuracy of the treatment. All competence domains were evaluated in relation China’s national clinical guidelines [7]. The detailed standards can be found in Supplementary file 1.

### Statistical analysis

We calculated the mean or proportion across all vignette interactions for each of our primary outcomes: ARQE, correct diagnosis, and correct treatment. To assess the correlates of these variables, we used ordinary least squares (OLS) regression for ARQE (continuous variable) and logistic regression for correct diagnosis and correct treatment (binary variables). For each outcome, we assessed correlations with a fixed set of facility-level and provider-level characteristics hypothesized to be related to provider competence.

We simulated system-level results by combining facility sorting behavior data with provider competence data from VCs and THCs to build a hypertension management chain that represented the entire rural system. We build this chain in three separate policy scenarios: free care seeking (Scenario 1), village clinics as the initial choice (Scenario 2), and township health centers as the initial choice (Scenario 3). For Scenario 1, we used the facility sorting behavior data collected by the household survey to determine which facility a patient would visit initially and then used hypertension treatment and referral data at the facility level to determine whether a patient would be treated correctly or to where the patient would be referred. For instance, if a patient initially decided to visit a VC, we used the correct treatment and referral data at the VC level (and, subsequently, the THC level) to calculate the probability of hypertension’s being correctly managed within the rural healthcare system. For Scenarios 2 and 3, we estimated the probability of correct hypertension management if all initial patient choices were to visit a VC or a THC, respectively. We then compare the probability of correct management between the three scenarios. All analyses were conducted using Stata V.14.0.

## Results

### Characteristics of providers and facilities

THC and VC providers differed significantly on a variety of provider-level characteristics (Table 1). THC providers were 43.0 years old, on average, 5 years younger than their VC counterparts (*p* < 0.001). THC providers tended to be more educated than were VC providers, as 20.9% of THC providers held at least a bachelor’s degree, compared to only 0.9% of VC providers (*p* < 0.001). Although VC providers had 24.9 years of medical experience, on average, compared to the 20.3 years of experience of THC providers (*p* < 0.001), only 6.4% of VC providers had practicing physician certificates, while 63.8% of THC providers did (*p* < 0.001). The average income of THC providers, 4361.9 yuan/month, was nearly two times that of VC providers, who made 2488.3 yuan/month (*p* < 0.001). Only 39.2% of THC providers participated in any physician training in the last year, whereas 64.3% of VC providers did the same (*p* < 0.001).

**Table 1:** Characteristics of providers and facilities at the township and village levels

Characteristics	THC Mean (*SD*) or % (*n*)	VC Mean (*SD*) or % (*n*)	*p-*value
Provider level			
Age (years)	43.0 (8.8)	48.0 (7.0)	< 0.001
Male (%)	58.7 (115)	69.1 (76)	0.07
Bachelor’s degree or higher (%)	20.9 (41)	0.9 (1)	< 0.001^a^
Medical experience (years)	20.3 (9.5)	24.9 (8.1)	< 0.001
Practicing physician certificate (%)	63.8 (125)	6.4 (7)	< 0.001
Income (yuan/month)	4361.9 (1187.9)	2488.3 (1088.7)	< 0.001
Work motivation (score)	48.9 (6.7)	49.9 (8.2)	0.27
Medical Training (frequency)	10.6 (12.0)	10.0 (7.7)	0.63
Lecture training (%)	95.4 (166)	98.1 (104)	0.71
NCD training in last year (frequency)	4.0 (4.6)	6.2 (4.9)	< 0.001
NCD training in last year (%)	39.2 (28.7)	64.3 (27.4)	< 0.001
Total	196	110	
Facility level			
Population in catchment areas (thousand)	35.2 (36.4)	1.78 (0.9)	< 0.001
Number of managed hypertension patients	1304.9 (650.3)	99.4 (58.8)	< 0.001
Number of full-time staff	42.6 (32.1)	1.2 (0.4)	< 0.001
Total	50	103	

Note. *a. Fisher’s exact probability was used to test the difference*

On a facility level, THCs served a larger population of patients than did VCs and were better staffed to do so. THC catchment areas serviced 35,200 residents, on average, a much larger number than that of VCs, at 1780 residents (*p* < 0.001). Similarly, THCs managed 1304 patients with hypertension, on average; VCs managed only 99.4 (*p* < 0.001). On average, THCs employed 42.6 full-time staff, while VCs employed only 1.2 (*p* < 0.001).

### Provider competence and correlates

**Fig. 1:**
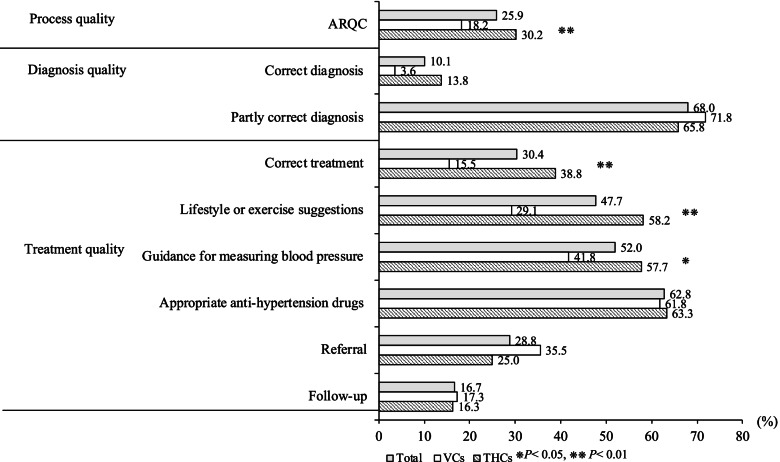
The comparison of provider competence between village clinics and township health centers. Note: ARQE is the average percentage of recommended questions and examinations

According to the analysis, the management of the healthcare needs of hypertension patients was poor throughout the rural healthcare system (Fig. 1). Across all interactions, the ARQE was only 25.9%. Only 31 of 306 interactions (10.1%) ended in a correct diagnosis. In the case of 208 interactions (68.0%), the vignette ended in a partially correct diagnosis. Finally, 94 of the 306 tested providers (30.4%) were able to provide the correct treatment. Breaking treatment into its individual factors, we found that 146 of 306 providers (47.7%) gave lifestyle and exercise suggestions, while 159 (52.0%) provided guidance for measuring blood pressure. A larger number of providers, 192 of 306 (62.8%), prescribed appropriate anti-hypertension drugs. In 88 of 306 interactions (28.8%), patients were referred to another facility, while in only 51 interactions (16.7%) did the provider request that the patient make a follow-up appointment.

Hypertension was managed more effectively at THCs than VCs, as THC providers scored significantly better than did VC providers on multiple measures of competence. The ARQE of THC providers was 30.2%, compared to 18.2% of VC providers (*p* < 0.01). Similarly, among the 196 THC providers, 76 (38.8%) provided the correct treatment, while only 17 of the 110 VC providers (15.5%) did the same (*p* < 0.01). In terms of individual treatment options, lifestyle and exercise suggestions were given by 114 of the 196 THC providers (58.2%). In comparison, only 32 of the 110 VC providers (29.1%; *p* < 0.01) gave lifestyle and exercise suggestions. Guidance for measuring blood pressure was given in 113 of the 196 THC interactions (57.7%), compared with 46 of the 110 VC interactions (41.8%, *p* < 0.05).

Provider competence in correct diagnosis and correct treatment was correlated with relatively few provider and facility characteristics (Table 2). Provider age was negatively correlated with correct diagnosis (OR = 0.85; 95% CI: 0.73, 0.98) and correct treatment (OR = 0.87, 95% CI: 0.80, 0.95). THC providers were 4.47 times (95% CI: 1.07, 18.67) more likely to give a correct diagnosis than those from VCs, while providers with more medical experience (OR = 1.12, 95%: 1.03, 1.21) were more likely to administer the correct treatment.

**Table 2:** Correlational analysis of provider’s correct diagnosis and treatment

Characteristic	Correct Diagnosis OR (95% CI)	Correct treatment OR (95% CI)
Age (years)	0.85*	0.87*
	(0.73, 0.98)	(0.80, 0.95)
Male (1 = yes)	1.10	1.64
	(0.46, 2.62)	(0.92, 2.95)
Bachelor’s degree or higher (1 = yes)	1.24	0.98
	(0.42, 3.68)	(0.43, 2.23)
Medical experience (years)	1.13	1.12*
	(0.99, 1.28)	(1.03, 1.21)
Practicing certificate (1 = yes)	0.72	1.86
	(0.28, 1.84)	(0.96, 3.60)
Income (yauan/month)	1.13	1.06
	(0.81, 1.58)	(0.83, 1.36)
Work motivation (score)	0.99	1.00
	(0.93, 1.05)	(0.96, 1.04)
THC provider (1 = yes)	4.47*	1.66
	(1.07, 18.67)	(0.68, 4.05)
Hypertension patients among the catchment area (%)	1.00	0.99
	(0.88, 1.14)	(0.91, 1.07)
NCD training among all types of training (%)	2.29	1.29
	(0.54, 9.66)	(0.48, 3.41)
Number of full-time staff	1.00	1.00
	(0.98, 1.01)	(0.99, 1.01)
Sample size	306	306

*p < 0.05

### Ability of the healthcare system to manage hypertension

**Fig. 2:**
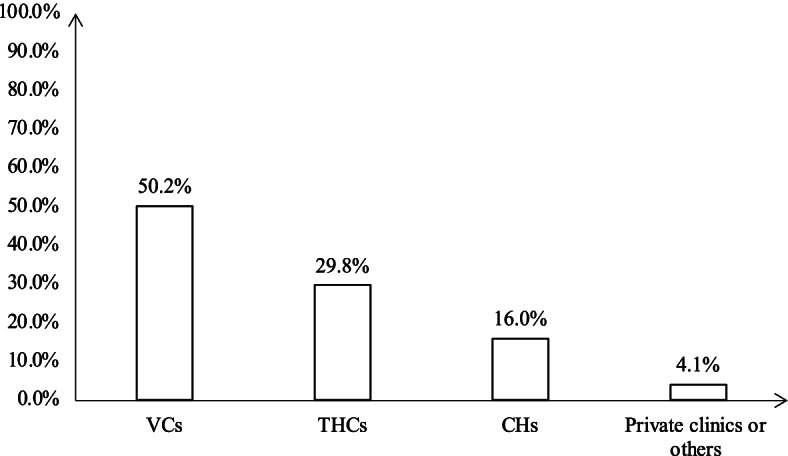
Sorting behaviors among adults with hypertension symptoms (*N* = 1526)

**Fig. 3:**
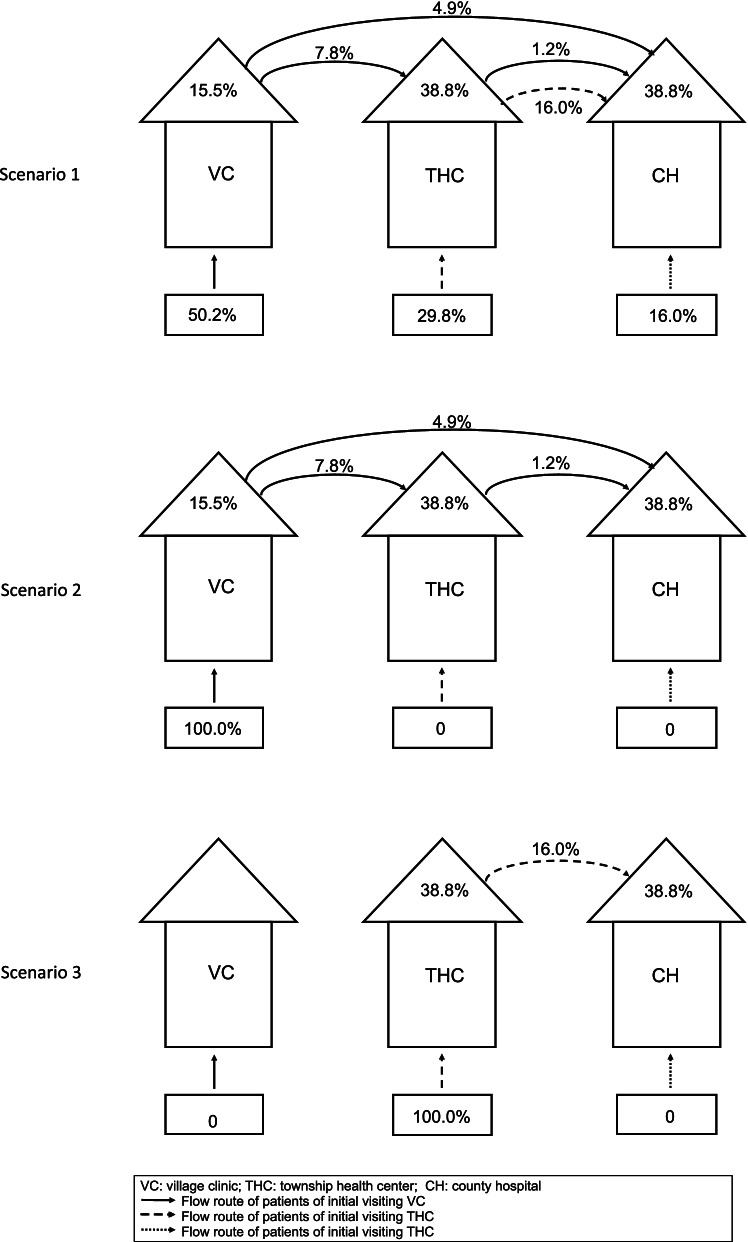
The probability of correct treatment in the rural healthcare system in three scenarios. Note: We assumed that CHs had the same probability of managing hypertension as did THCs. This may underestimate the ability of the entire rural healthcare system, but it does not influence the comparison of the three scenarios

To demonstrate system-level quality, we combined provider competence, facility sorting behavior and referral data (Fig. 2 and Additional Table 3) to produce a model that displays the management of hypertension at each facility and any subsequent patient referral to higher-level facilities in several policy scenarios (Fig. 3). The collected facility sorting behavior indicated that 50.2% of hypertension patients visited VCs initially, while 29.8% visited THCs, and 16.0% went directly to a CH. Using the data from Fig. 1 and Additional Table 3, we attribute correct management of hypertension to be 15.5% at VCs and 38.3% for THCs and CHs. Of patients whose symptoms were not managed at a VC, 7.8% were given a referral to a THC, and 4.9% were referred to a CH directly. Among the 7.8% of patients who transferred to a THC from a VC, 4 people were subsequently referred to a CH, accounting for 1.2% of referrals. For patients who visited a THC initially, 16% were referred to a CH.

Using the model and our assumptions, we calculate the probability that the average rural hypertension patient will receive the correct treatment under three policy scenarios (Table 3). In Scenario 1, we use the status quo sorting behaviors (patients’ freely selecting into facilities) and find that the probability of correct management throughout the rural system was equal to 29.8%. In this scenario, 7.8% of patients were correctly managed at VCs; 13.1% of patients, at THCs; and 9.0% of patients, at CHs. In Scenario 2, we assumed that all patients first visited a VC, as is encouraged by China’s current healthcare reform policy, and kept management and referral probabilities unchanged. The probability of correct management for patients in Scenario 2 was 20.5%. To better compare the first two scenarios, in Scenario 3, we assumed that all patients initially visited THCs, again keeping management and referral probabilities unchanged, finding that the probability of correct management was equal to 45.0%. Detailed calculation information can be found in Table 3.

**Table 3:** Calculation of the probability of correct management in the rural healthcare system

Scenario	VCs	THCs	CHs	Total
Scenario 1				
1. Initial visit	50.2%*15.5%	29.8%*38.8%	16.0%*38.8%	25.6%
2. Referral from VC	–	50.2%*7.8%*38.8%	50.2%*4.9%*38.8%	2.5%
3. Referral from THC^a^	–	–	50.2%*7.8%*1.2%*38.8%+ 29.8%*16.0%*38.8%	1.8%
Total	7.8%	13.1%	9.0%	**29.8%**
Scenario 2				
1. Initial visit	100%*15.5%	0.0%	0.0%	15.5%
2. Referral from VC	–	100%*7.8%*38.8%	100%*4.9*38.8%	4.9%
3. Referral from THC	–	–	100%*7.8%*1.2%*38.8%	0.0%
Total	15.5%	3.0%	5.0%	**20.5%**
Scenario 3				
1. Initial visit	0.0%	100%*38.8%	0.0%	38.8%
2. Referral from VC	–	–	–	
3. Referral from THC	–	–	100%*16.0%*38.8%	6.2%
Total	0.0%	38.8%	6.2%	**45.0%**

Note: Based on the appointment data of the patients, three groups were constructed within each scenario: initial visits, referral from a VC, and referral from a THC. We did not collect data on CHs, and, thus, we assume that CHs had correct treatment rates equal to those of the THCs, which is a conservative estimate, as CH providers have been shown to have higher rates of correct treatment than do THC providers when managing disease s[7]. We combined facility-level data (Table 2) and facility sorting behaviors (Supplementary Fig. 1) to calculate the probability of correct management on a facility level. The probability of correct management within the healthcare system was calculated by summing the results of the three levels

a. This group included two groups of patients, the first of which visited a VC initially, then was referred to a THC, and, finally, was referred to a CH. The second group visited a THC initially and was then referred to a CH. Correct management for the first group was calculated as follows: 50.2% of patients visited a VC initially, had a 7.8% probability of being referred to a THC, and a 1.2% probability of then being further referred to a CH and, from there, of having a 38.8% probability of correct management. Correct management for the second group was calculated as follows: 29.8% of residents visited a THC initially, had a 16.0% probability of being referred to a CH and, from there, having a 38.8% probability of correct management. Bold indicates the total probability for correct management in each policy scenario.

## Discussion

Our study uses clinical vignettes to evaluate competence in management of patients symptomatic of hypertension among healthcare providers in rural China. Our analysis finds that rates of correct diagnosis and treatment of patients symptomatic of hypertension among the rural providers in the sample were generally low. Large gaps between VC and THC providers also are apparent, as providers from THCs were more competent not only during the consultation process but also in treatment, providing guidance in lifestyle management and blood pressure measurement more frequently. Provider competence also varies with provider-level characteristics; younger and more experienced providers display higher competence. By combining provider competence and facility sorting behavior, we are able to model hypertension management in the rural system as a whole. Using this model, we find that free selection of treatment facilities leads to poor management at the system level. This management worsens when patients choose to visit only VCs and improves when patients choose to visit only THCs.

The low detection and management rates are consistent with the literature in China and internationally. In rural China, previous research on the quality of health care has found that the competence of primary care providers is relatively low [28]. This is also a problem internationally, as low- and middle-income countries (LMICs) also struggle with insufficient competence in primary care—especially in poorer subpopulations [29]. Overall, health providers from LMICs demonstrate poor quality of clinical practice, often following fewer than half of recommended procedures [30]. Due to this poor competence, future healthcare improvement measures should be focused on primary care providers in rural China and other LMIC settings.

The gap between VC and THC providers also is consistent with past studies that have noted concerns in the ability of village clinicians to manage chronic diseases [24, 31]. In rural China, shortcomings in the diagnostic competence of VC providers have hindered the management of other NCDs, such as unstable angina [31]. These results also are consistent with findings from other LMICs. In India, provider competence varies between facilities to a striking degree [32]. Given the existing gap in competence at the lowest levels of the healthcare system and the importance of controlling chronic diseases [33], future research and policy implementation are needed to narrow the competence gap between rural providers.

Variations in the ability of rural providers to detect and manage patients symptomatic of hypertension have been identified in previous research [34, 35], in line with our findings. In rural China, provider competence in hypertension treatment and prevention was found to vary by age, gender, and work experience [36, 37]. Unlike other studies [38, 39], however, we did not find that NCD training contributed to provider competence in diagnosis or treatment. Our results indicate that the current NCD training methods may not improve provider competence in hypertension management. Further research is needed to explore the potential reasons for these NCD training shortcomings and to produce effective training methods for primary healthcare providers.

Combining provider competence and patients’ facility sorting behavior of local residents into a single model, as we have done in three policy scenarios, has been used in previous research to analyze system-level competence in disease management and healthcare utilization [15, 21, 40]. The low competence that we find in Scenario 1, in which patients freely select which facility to visit, reveals that freely selecting an initial facility may not contribute to the effective management of hypertension. This should be taken into consideration when designing policy for the rural healthcare system, as patients are not able to select the facility that provides them with the best outcome.

Scenario 2, in which patients first visit only VCs, may, unfortunately, become a reality. This scenario is encouraged by a recent nationwide policy that pushes residents to first visit the lowest-level facility within the system hierarchy, which works well in urban areas but not in rural settings [41]. The literature has shown that VC providers present multiple disadvantages (which result in poorer outcomes) as the first point of contact for rural residents [42, 43]. This may have considerable negative impacts, as low competence in hypertension management leads to lower control rates, dropping to lower than 5% in some rural areas [44, 45]. Previous studies have shown that inappropriate clinical practice, staff shortages, and aging providers are common challenges among these rural facilities [46, 47]. Without strengthening the competence of VC providers, recent changes in the health policy may not contribute to effective hypertension management and may even negatively affect patient health outcomes.

The higher systemic competence in hypertension management found in Scenario 3, in which patients first visit only THCs, is promising and should be a consideration in future policy changes. This may be because THC providers had higher competence in managing hypertension than those from VCs, as VC providers have been identified as lacking skills and qualifications [48]. Previous research also has suggested that THCs may be more appropriate facilities for first-contact care [49]. Therefore, new interventions designed to reduce costs for patients at the THC level, such as telemedicine [50], should be consider so as to increase the prevalence of first-contact with THC providers.

### Limitations

Our study has several limitations. First, the clinical vignette method cannot be used to measure actual clinical practice when providers are faced with real first-visit patients, and, thus, our results may overestimate provider competence in managing patients symptomatic of hypertension. Second, we did not measure the competence of CH providers, assuming, instead, that their competence is the same as that of THC providers. This assumption was used only to compare the effects of the three policy scenarios and, thus, does not influence our primary analysis of VC and THC providers. Further research should include CH providers and evaluate their competence. Third, this study does not examine the broad array of factors that may inform patients’ initial choice of facility, including quality, price, location, and others. Further research of these factors can help to inform policy changes in hypertension management based on patient behavior.

## Conclusion

Providers from the rural primary healthcare system do not yet have enough competence to manage patients symptomatic of hypertension to an acceptable degree and, thus, cannot overcome the considerable challenge of this highly prevalent chronic disease in rural China. National policy that advises residents to first visit the lowest levels in the healthcare system may be constraining the system in addressing this challenge. Research to improve diagnosis and treatment competence for hypertension and design effective health policy is needed.

## Supplementary Information

**Additional file 1.**

## Data Availability

The datasets generated and/or analysed during the current study are available from the corresponding author, Huan Zhou, on reasonable request.

## Abbreviations

VC: Village clinic
THC: Township health center
CH: County hospital
NCD: Non-communicable disease
LMIC: Low- and middle-income country
ARQE: Average percentage of recommended and essential questions and examinations
OLS: Ordinary least squares

## References

[1] Naghavi M, Abajobir AA, Abbafati C, Abbas KM, Abd-Allah F, Abera SF (2017). Global, regional, and national age-sex specific mortality for 264 causes of death, 1980–2016: a systematic analysis for the global burden of disease study 2016. Lancet..

[2] Marczak L, Williams J, Loeffler M (2018). for the For the Institute for Health Metrics and Evaluation. Global Deaths Attributable to High Systolic Blood Pressure, 1990-2016. JAMA.

[3] Lu J, Lu Y, Wang X, Li X, Linderman GC, Wu C (2017). Prevalence, awareness, treatment, and control of hypertension in China: data from 1·7 million adults in a population-based screening study (China PEACE million persons project). Lancet..

[4] Zhang Y, Tang W, Zhang Y, Liu L, Zhang L. Effects of integrated chronic care models on hypertension outcomes and spending: a multi-town clustered randomized trial in China. BMC Public Health. 2017;17. 10.1186/s12889-017-4141-y PMC5346199 28284202

[5] Yip WC-M, Hsiao WC, Chen W, Hu S, Ma J, Maynard A (2012). Early appraisal of China’s huge and complex health-care reforms. Lancet..

[6] Li X, Lu J, Hu S, Cheng K, De Maeseneer J, Meng Q (2017). The primary health-care system in China. Lancet..

[7] Writing Group of 2018 Chinese Guidelines for the Management of Hypertension, Chinese Hypertension League, Chinese Society of Cardiology, Chinese Medical Doctor Association Hypertension Committee, Hypertension Branch of China International Exchange and Promotive Association for Medical and Health Care, Hypertension Branch of Chinese Geriatric Medical Association. Chinese guidelines for prevention and treatment of hypertension (2018 Revised version). Chinese Journal of Cardiovascular Medicine 2019;24:24–56.

[8] Li Y, Yang L, Wang L, Zhang M, Huang Z, Deng Q (2017). Burden of hypertension in China: a nationally representative survey of 174,621 adults. Int J Cardiol.

[9] Li H, Meng Q, Sun X, Salter A, Briggs NE, Hiller JE (2010). Prevalence, awareness, treatment, and control of hypertension in rural China: results from Shandong Province. J Hypertens.

[10] Chinese Medical Association, Chinese medical journals publishing house, Chinese Society of General Practice, EditorialBoardofChineseJourna lof GeneralPractitioners. Guidelines for primary diagnosis and treatment of hypertension (2019). Chinese J Gen Pract. 2019;04:301–13.

[11] Zhang D, Pan X, Li S, Liang D, Hou Z, Li Y (2017). Impact of the National Essential Public Health Services Policy on hypertension control in China. Am J Hypertens.

[12] Xia L, Ning N, Hao Y, Hong S, Gao L, Jiao M, et al. Health literacy in rural areas of China: hypertension knowledge survey. Int J Environ Res Public Health. 2013. 10.3390/ijerph10031125. 10.3390/ijerph10031125 PMC3709308 23507738

[13] Li X, Cai L, Wang GY, Fan LM, Golden AR. Socioeconomic and lifestyle determinants of prevalence of hypertension among the elderly in rural Southwest China: a structural equation modeling approach. Int J Equity Health. 2020. 10.21203/rs.3.rs-32366/v1. 10.1186/s12872-021-01885-y PMC7851929 33530935

[14] Xue J, Chen S, Bogner HR, Tang W, Li L, Conwell Y (2017). The prevalence of depressive symptoms among older patients with hypertension in rural China. Int J Geriatr Psychiatry.

[15] Sylvia S, Xue H, Zhou C, Shi Y, Yi H, Zhou H (2017). Tuberculosis detection and the challenges of integrated care in rural China: a cross-sectional standardized patient study. PLoS Med.

[16] Abolfotouh MA, Soliman LA, Abolfotouh SM, Raafat M (2011). Knowledge and practice of PHC physicians toward the detection and Management of Hypertension and Other CVD risk factors in Egypt. Int J Hypertens.

[17] Masi C, Hamlish T, Davis A, Bordenave K, Brown S, Perea B (2012). Using an established telehealth model to train urban primary care providers on hypertension management: telehealth training to manage hypertension. J Clin Hypertens (Greenwich).

[18] Ahmad N, Khan AH, Khan I, Khan A, Atif M (2018). Doctors’ knowledge of hypertension guidelines recommendations reflected in their practice. Int J Hypertens.

[19] Wang WH, Zhao D, Zeng ZC, Jia YN, Liu Y, Zhu XP. A cross-sectional study on knowledge and the ability of hypertension treatment among physicians in district and community hospitals. Chinese J Epidemiol. 2003. 14761620

[20] Chen Q, Zhang X, Gu J, Wang T, Zhang Y, Zhu S (2013). General practitioners’ hypertension knowledge and training needs: a survey in Xuhui district. Shanghai BMC Fam Pract.

[21] Peabody JW, Tozija F, Muñoz JA, Nordyke RJ, Luck J (2004). Using vignettes to compare the quality of clinical care variation in economically divergent countries. Health Serv Res.

[22] Veloski J. Clinical vignette-based surveys: a tool for assessing physician practice variation. Am J Med Qual. 2005. 10.1177/1062860605274520. 10.1177/1062860605274520 15951521

[23] Gidengil CA, Linder JA, Beach S, Setodji CM, Hunter G, Mehrotra A. Using clinical vignettes to assess quality of Care for Acute Respiratory Infections. Inquiry. 2016;53. 10.1177/0046958016636531 PMC4840477 27098876

[24] Windak A, Gryglewska B, Tomasik T, Narkiewicz K, Yaphe J, Grodzicki T (2010). The competence of primary care doctors in the investigation of patients with elevated blood pressure: results of a cross-sectional study using clinical vignettes. J Eval Clin Pract.

[25] Humbert X, Fedrizzi S, Touzé E, Alexandre J, Puddu P-E. White-coat hypertension: management and adherence to guidelines by European and Canadian GPs. A cross-sectional clinical vignette study. BJGP Open. 2019;3. 10.3399/bjgpopen19X101664 PMC6995860 31581110

[26] Sichuan provincial bureau of statistics. Sichuan Stat Yearbook 2021. 2022. http://tjj.sc.gov.cn/scstjj/c105855/nj.shtml. Accessed 29 Apr 2022.

[27] Gagné M, Forest J, Gilbert M-H, Aubé C, Morin E, Malorni A (2010). The motivation at work scale: validation evidence in two languages. Educ Psychol Meas.

[28] Yip W, Fu H, Chen AT, Zhai T, Jian W, Xu R (2019). 10 years of health-care reform in China: progress and gaps in universal health coverage. Lancet..

[29] Du S, Cao Y, Zhou T, Setiawan A, Thandar M, Koy V (2019). The knowledge, ability, and skills of primary health care providers in SEANERN countries: a multi-national cross-sectional study. BMC Health Serv Res.

[30] Kruk ME, Gage AD, Arsenault C, Jordan K, Leslie HH, Roder-DeWan S (2018). High-quality health systems in the sustainable development goals era: time for a revolution. Lancet Glob Health.

[31] Guo W, Sylvia S, Umble K, Chen Y, Zhang X, Yi H (2020). The competence of village clinicians in the diagnosis and treatment of heart disease in rural China: a nationally representative assessment. Lancet Regional Health - Western Pacific.

[32] Das J, Mohpal A (2016). Socioeconomic status and quality of care in rural India: new evidence from provider and household surveys. Health Aff (Millwood).

[33] Beaglehole R, Epping-Jordan J, Patel V, Chopra M, Ebrahim S, Kidd M (2008). Improving the prevention and management of chronic disease in low-income and middle-income countries: a priority for primary health care. Lancet..

[34] Yan LD, Chirwa C, Chi BH, Bosomprah S, Sindano N, Mwanza M (2017). Hypertension management in rural primary care facilities in Zambia: a mixed methods study. BMC Health Serv Res.

[35] Laar AK, Adler AJ, Kotoh AM, Legido-Quigley H, Lange IL, Perel P (2019). Health system challenges to hypertension and related non-communicable diseases prevention and treatment: perspectives from Ghanaian stakeholders. BMC Health Serv Res.

[36] Zhao Y, Li Q, Dang S, Chen Y, Cao L, Yang R, et al. Team structure and hypertension treatment and prevention ability of village docotors in rural areas of Hanzhong, Shaanxi province. Chinese General Practice. 19:1955–9.

[37] Li N, Diao W, Mu H, Xing L, Yu L. Evaluation capability of hypertension treatment by rural doctors from six counties of Liaoning Province. Chinese. Gen Pract. 2012.

[38] Das J, Chowdhury A, Hussam R, Banerjee AV. The impact of training informal health care providers in India: a randomized controlled trial. Science. 2016;354. 10.1126/science.aaf7384 27846471

[39] Malan Z, Mash R, Everett-Murphy K (2015). Qualitative evaluation of primary care providers experiences of a training programme to offer brief behaviour change counselling on risk factors for non-communicable diseases in South Africa. BMC Fam Pract.

[40] Fe E, Powell-Jackson T, Yip W (2017). Doctor competence and the demand for healthcare: evidence from rural China. Health Econ.

[41] Zhou Z, Zhao Y, Shen C, Lai S, Nawaz R, Gao J (2021). Evaluating the effect of hierarchical medical system on health seeking behavior: a difference-in-differences analysis in China. Soc Sci Med.

[42] Li X, Krumholz HM, Yip W, Cheng KK, Maeseneer JD, Meng Q (2020). Quality of primary health care in China: challenges and recommendations. Lancet..

[43] Babiarz KS, Miller G, Yi H, Zhang L, Rozelle S (2012). China’s new cooperative medical scheme improved finances of township health centers but not the number of patients served. Health Aff (Millwood).

[44] Li Y, Wang L, Feng X, Zhang M, Huang Z, Deng Q (2018). Geographical variations in hypertension prevalence, awareness, treatment and control in China: findings from a nationwide and provincially representative survey. J Hypertens.

[45] Xing L, Liu S, Tian Y, Jing L, Ren G, Dong Y (2019). Trends in status of hypertension in rural Northeast China: results from two representative cross-sectional surveys, 2013–2018. J Hypertens.

[46] Li X, Cochran C, Lu J, Shen J, Hao C, Wang Y (2015). Understanding the shortage of village doctors in China and solutions under the policy of basic public health service equalization: evidence from Changzhou. Int J Health Plann Manag.

[47] Dyar OJ, Yang D, Yin J, Sun Q, Lundborg CS (2020). Variations in antibiotic prescribing among village doctors in a rural region of Shandong province, China: a cross-sectional analysis of prescriptions. BMJ Open.

[48] Hu D, Zhu W, Fu Y, Zhang M, Zhao Y, Hanson K (2017). Development of village doctors in China: financial compensation and health system support. Int J Equity Health.

[49] Liu Y, Kong Q, de Bekker-Grob EW (2019). Public preferences for health care facilities in rural China: a discrete choice experiment. Soc Sci Med.

[50] Kvedar J, Coye MJ, Everett W (2014). Connected health: a review of technologies and strategies to improve patient care with telemedicine and telehealth. Health Aff.

