# Supplementary material for: Provider competence in hypertension management and challenges of the rural primary healthcare system in Sichuan province, China: a study based on standardized clinical vignettes
Source: BMC Health Serv Res. 2022 Jul 1;22:849. doi: 10.1186/s12913-022-08179-9 (PMC9248120; doi:10.1186/s12913-022-08179-9)
Supplement: Supplementary file 1 — Additional file 1. [file 12913_2022_8179_MOESM1_ESM.pdf]

Additional Text A1

The research team developed a standardized case script following several rounds of consultation with hypertension specialists. Local hypertension prevention and management authorities also were included in the consultation to adapt the script to a local context. This standardized case script outlined the scenario that enumerators simulated during the vignette. Each vignette was conducted by two enumerators. One enumerator assumed the role of “mock patient,” and the other assumed the role of “facilitator.” The facilitator stated the instructions to the providers, documented the interaction, and provided additional information that the patient might not know but that the provider would determine if he or she actively solicited it, such as the results of tests or examinations. To begin the vignette, the facilitator informed the doctor that a 65-year-old male patient was visiting the clinic. The mock patient then told the doctor about his problem with an opening statement (“Doctor, I have headache, and my face is a little hot”). Providers were then asked to proceed as they would with a real patient and were told that the patient would answer any questions asked and comply with any instructions given. During the interaction, the facilitator documented the provider’s questions, diagnostic examination requests, stated diagnosis, treatment prescribed (drugs or patient education/instructions), and whether the provider referred the patient to another provider or facility.

Hypertension cases

Observer: “A 65-year-old man came to you and asked you to take a look at his symptoms.”

Patient: “Doctor, I have a headache, and my face is a little hot.”

Story background

Male patient: Zhao Jianguo, 65 years old. Wife: Zhang Lixia, female, 62 years old. They have three sons and two daughters.

Rule: Doctors don’t ask, patients don’t say. This encapsulates the aim of having all enumerators strictly follow our script, so as not to provide any additional information that could influence the consultation process, diagnosis, and treatment results.

Main story

1. “Since last month, I have been having headaches. This has never happened before.”
2. “I have had a headache all day, and the back of my head hurts. The pain is dull, and a little heavy. Sometimes my face gets a little hot when I have a headache. My face gets hot once every day or two, lasting for half an hour to an hour.”
3. Blood pressure: systolic blood pressure 155 mmHg, diastolic blood pressure 95 mmHg.
4. Patient height is 1.7 meters, and weight is 76 kg. Waist circumference is 70 cm; heart rate/pulse rate is 80 beats per minute.
5. Blood sugar is normal, blood lipids have not been checked, and no other abnormal symptoms are present. There are no other diseases in the body.
6. “I usually eat a normal amount, and about the same as other local families. I don’t drink alcohol, in general, and drink only 50 to 100 grams of liquor when guests come to the house.”
7. “My work is usually farming. The work is not heavy, not very tiring, and doesn’t do much harm to my body.”
8. Everyone in the family is in good health, and there are no major health problems.

Additional Text A2

The consultation process was evaluated by comparing the questions and physical examinations that providers used in the interaction to a standard checklist that provided China’s

national clinical guidelines. The national guidelines contained 22 recommended questions (including 5 essential questions), and 8 recommended examinations (including 2 essential examinations), as seen in Table 1.

Additional Table 1. Recommended items and essential items for questions and examinations

Item No.	Recommended item (consultation)	Essential item
1	Onset of problems	Yes
2	Headache duration	Yes
3	Pain location	Yes
4	Type of the pain	Yes
5	What makes the headache worse or better?	No
6	Pain radiation	No
7	Temperature	No
8	Weakness of the arms or legs	No
9	Vomiting/nausea	No
10	Dizziness	No
11	Diet	No
12	Alcohol use	No
13	Smoking habits	No
14	Diabetes	No
15	Heart problems	No
16	Hyperlipidemia	No
17	Kidney problems	No
18	Bronchus problems	No
19	Sleep	No
20	Occupation	No
21	Family history	No
22	Age	Yes
**Item No.**	**Recommended item (examinations)**	**Essential item**
1	Blood pressure	Yes
2	Physical examination (height, weight and waistline)	Yes
3	Temperature	No
4	Heart rate/pulse rate	No
5	Routine urine test	No
6	Routine blood test	No

Item No.	Recommended item (consultation)	Essential item
7	Blood biochemistry	No
8	Electrocardiogram	No

The consultation process quality indicator was defined as the average percentage of recommended questions and examinations (ARQE). This indicator was calculated for each provider as the number of recommended questions and examinations mentioned in the interaction (n_1) divided by the total number of questions and examinations recommended by national guidelines (n_1). The formula is as follows:

$$ARQE = \frac{n_1}{n_2}$$

A correct diagnosis was achieved if the providers diagnosed the patient with a "Medium risk of hypertension" or "Hypertension stage 1" in the interaction. A correct treatment was achieved by meeting either of two conditions without prescribing harmful medication, such as a large infusion of normal saline: (1) The provider gave his or her patients suggestions for diet, lifestyle, and exercise and guidance to test blood pressure every day; or (2) Condition 1 plus the prescription of appropriate antihypertensive drugs (detailed treatment conditions are listed in Table 2). Finally, any referral to another healthcare facility and whether any follow-up appointment was requested by the provider was recorded.

Additional Table 2. Standards for hypertension

Diagnosis standard		
Correct	Partially correct	Wrong
Medium risk of hypertension<br>Stage 1	Hypertension	Other
	Low risk of hypertension<br>Hypertension Stage 2<br>Essential hypertension	Secondary hypertension
Treatment standard		
Either 1 or 2:	Any of 1–5:	Other

1. A+B<br>2. A+B+C	1. Only A<br>2. Only B<br>3. Only C<br>4. A+C<br>5. B+C

Note:
A: Give diet, lifestyle, and exercise advice
B: Instruct the patient in how to check blood pressure every day
C: One or more of the following four drugs:
(1) Angiotensin converting enzyme inhibitor (ACEI) or angiotensin receptor blocker (ARB)
(2) Beta blockers
(3) Long-acting calcium channel blockers (CCBs)
(4) Thiazide diuretics

Additional Table 3. Patient referral data from primary care providers

Referral direction	Numbers (n)	Percent (%)
VCs→THCs	24	7.8%
VCs→CHs	15	4.9%
THCs→CHs#	4	1.2%
THCs→CHs	49	16.0%

Patient's' initial choice is VCs. When they were referred to THCs, some of them will be referred to CHs.

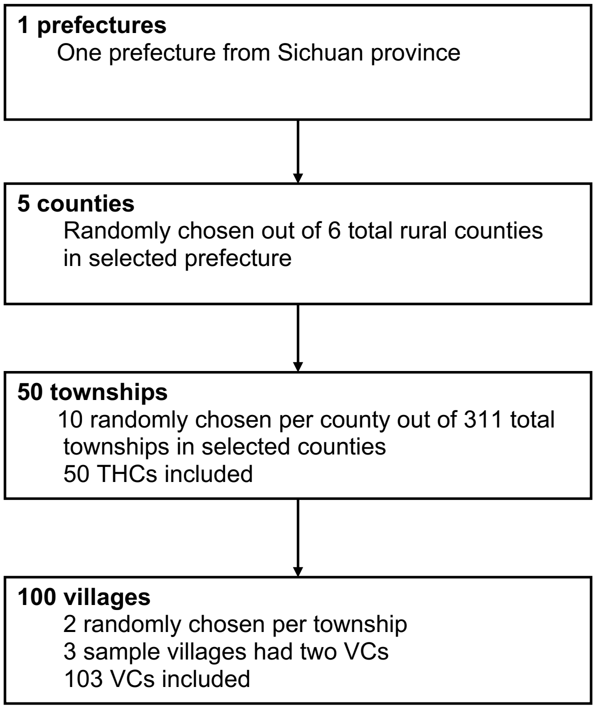

Additional Figure 1 STROBE follow chart of the sampling process
